# Comparison of homocitrulline and carbamylated albumin as biomarkers of carbamylation reactions in hemodialyzed patients

**DOI:** 10.1007/s00726-023-03306-0

**Published:** 2023-08-02

**Authors:** Aurelie Lenglet, Stéphane Jaisson, Philippe Gillery, Souleiman El Balkhi, Sophie Liabeuf, Ziad A. Massy

**Affiliations:** 1 UM7517, MP3CV Laboratory, CURS, Faculty of Pharmacy, Jules Verne University of Picardie, Amiens, France; 2 Pharmacy Division, Amiens University Medical Center, Amiens, France; 3 University of Reims Champagne-Ardenne, CNRS, MEDyC Unit UMR 7369, Reims, France; 4 Biochemistry Department, Reims University Medical Center, Reims, France; 5 INSERM, IPPRITT, U1248, 87000 Limoges, France; 6 Division of Nephrology, Ambroise Paré Hospital and Paris Ile de France Ouest University, 9 Avenue Charles de Gaulle, 92104 Boulogne Billancourt Cedex, France; 7 INSERM U-1018, Centre de recherche en épidémiologie et santé des populations (CESP), Paris-Saclay University (PSU) and University of Paris Ouest-Versailles-Saint-Quentin-en-Yvelines (UVSQ), Equipe 5, Villejuif, France

**Keywords:** Carbamylation, Homocitrulline, End-stage kidney disease

## Abstract

To describe the association between levels of homocitrulline (HCit) and the degree of albumin carbamylation in a cohort of hemodialyzed patients. Plasma total and protein-bound HCit concentrations in samples from hemodialyzed patients included in NICOREN trial were determined by LC–MS/MS at baseline and after 24 weeks of treatment with either sevelamer or nicotinamide. HCit concentrations at all timepoints and in both groups were positively and significantly correlated with the degree of albumin carbamylation. Plasma concentrations of total HCit, protein-bound HCit and carbamylated albumin did not decrease after 24 weeks of treatment with either sevelamer or nicotinamide. The present results demonstrate that plasma total and protein-bound HCit concentrations were closely associated with albumin carbamylation in hemodialyzed patients. Therefore, total and protein-bound HCit concentrations might be valuable biomarkers of the overall intensity of protein carbamylation in this context. Given the less complex and time-consuming analytical methods required, these markers should be favored in future clinical studies of carbamylation reaction.

## Introduction

The formation of carbamylation-derived products (CDPs) is observed in patients with chronic kidney disease (CKD) because urea accumulates in the body. Importantly, CDPs are strong predictors of mortality in CKD patients (Berg et al. 2013; Koeth et al. 2013) and an elevated risk of coronary artery disease, future myocardial infarction or stroke (Wang et al. 2007; Tang et al. 2013). A recent clinical study of a large cohort (*n* = 3111 patients) showed that higher level of protein carbamylation is an independent risk factor for developing end-stage kidney disease (ESKD) and significant adverse clinical outcomes (cardiovascular complications) (Kalim et al. 2023). Homocitrulline (HCit, formed by isocyanic acid covalent binding to the Ɛ-NH_2_-group of lysine residue side chain) is the best-characterized CDP (Kraus et al. 1994) and can reflect the average carbamylation rate of all carbamylated proteins. Indeed, all proteins can be targeted by isocyanic acid, and individual carbamylated proteins (such as albumin, collagen, erythropoietin, hemoglobin, low-density lipoprotein, and high-density lipoprotein) can be specifically quantified and thus also serve as biomarkers. We previously confirmed the presence of three carbamylated sites containing the peptide sequence LVRP on albumin in hemodialyzed patients (Lenglet et al. 2021). Although albumin is the most abundant serum protein, however, its carbamylation rate depends on many factors (such as its half-life) and does not reflect the carbamylation rate of other proteins—the contribution of which should not be overlooked. In contrast, HCit offers a broader overview of carbamylation. Moreover, the link between carbamylated albumin and HCit has not previously been evaluated in patients with ESKD. Hence, the objectives of the present study (an ancillary analysis of the NICOREN trial) were to (i) assess the putative link between HCit concentrations and the degree of albumin carbamylation in hemodialyzed patients, and (ii) determine whether or not treatment with sevelamer or nicotinamide was associated with changes in plasma HCit concentrations.

## Materials and methods

The NICOREN multicenter, open-label, randomized study was designed to examine the non-inferiority and safety of nicotinamide vs. sevelamer in chronic hemodialysis patients. In this ancillary analysis of the NICOREN study, plasma samples were available for 45 patients: 21 patients in the sevelamer group and 24 patients in the nicotinamide group. Serum urea concentrations were assayed in an on-site biochemistry laboratory, using standard techniques. After enrolment, samples of serum and plasma were immediately frozen at − 80 °C in the Biobanque de Picardie biological resource center (Amiens, France) prior to carbamylated protein measurements at the end of the study. Protein carbamylation was evaluated in terms of total and protein-bound HCit using LC–MS/MS (as described previously by Jaisson et al.) and expressed in µmol of HCit per mole of lysine (Jaisson et al. 2023). In 45 control subjects (i.e. individuals with no kidney disease and a serum creatinine concentration below 100 µmol/L), the median [interquartile range (IQR)] HCit concentration was 140 µmol/mol lysine [100–170] (Jaisson et al. 2015). Albumin carbamylation was measured using multiple reaction monitoring and MS/MS mass transitions and was quantified as the area under the curve (AUC). The degree of carbamylation (in %) was calculated as the AUC for the carbamylated peptide divided by the AUC for the non-carbamylated peptide. The most frequent carbamylation sites were in the **KQTA**LVELVK peptide (referred to henceforth as KQTA and containing lysine 549) and the **LVRP**EVDVMCTAFHDNEETFLKK peptide (referred to as LVRP) (Lenglet et al. 2021).

### Statistical analysis

Results were expressed as the mean ± standard deviation (SD), median [IQR] or frequency (percentage), as appropriate. The normality of the distribution of quantitative variables was checked with the Shapiro–Wilk test. The study population was divided into two treatment groups. Potential intergroup differences in mean values were evaluated in an analysis of variance. Comparisons of more than two mean values were assessed in a two-way analysis of variance with repeated measures. If a significant difference was found, Tukey’s post hoc test for multiple comparisons was used to confirm the result. Spearman’s correlation coefficient was calculated as a guide to the strength of associations. The threshold for statistical significance was set to *p* < 0.05. All statistical analyses were performed using SPSS software (version 18.0, SPSS Inc., Chicago, IL, USA) for Windows (Microsoft Corp, Redmond, WA, USA).

## Results

Forty-five patients (males: 68%; mean ± SD age: 65 ± 13; prevalence of diabetes: 44%) were included. The patients’ demographic, clinical and laboratory data are summarized in Table 1.

**Table 1:** Baseline demographic, clinical and laboratory data in the NICOREN cohort of patients with ESKD

Patients with ESKD (*n* = 45) Baseline	
Demographic variables	
Age (years)	65.3 ± 12.7
Men, *n* (%)	31 (68)
Sevelamer randomization group *n* (%)	21 (47)
Clinical variables	
BMI (kg/m^2^)	27.3 ± 4.9
History of CVD *n* (%)	26 (58)
Diabetes mellitus (%)	20 (44)
Dialysis dose (*K*_t_/*V*_urea_)	1.28 ± 0.3
Predialysis systolic BP (mmHg)	142 ± 25
Laboratory variables	
Serum C-reactive protein (mg/L)	4.2 [3.0–9.9]
Plasma urea (mmol/L)	24.5 ± 7.6
Plasma total homocitrulline (µmol/mol lysine)	624 [509–788]
Plasma protein-bound homocitrulline (µmol/mol lysine)	505 [393–603]
Degree of LVRP carbamylation (%)	1.5 [1.2–2.1]
Degree of KQTA carbamylation (%)	28.1 [21.8–33.4]

Quantitative variables are quoted as the mean ± SD or (for those with a non-Gaussian distribution) the median [IQR], and qualitative variables are quoted as the frequency (percentage). The degree of carbamylation (in %) was calculated as the AUC for the carbamylated peptide divided by the AUC for the non-carbamylated peptide

*CVD* cardiovascular disease, *BMI* body mass index, *LVRP and KQTA* the two albumin carbamylation sites

**Fig. 1:**
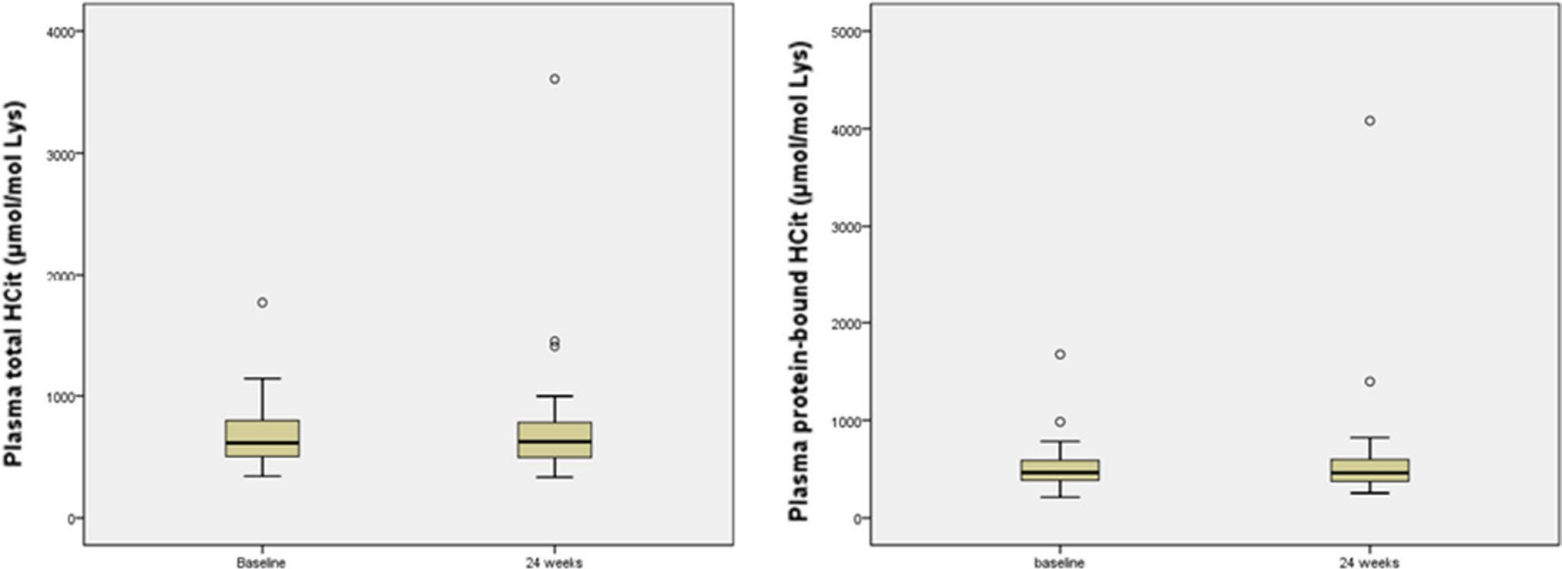
Baseline and 24-week plasma concentrations of total and protein-bound homocitrulline. *HCit* homocitrulline, *Lys* lysine

At baseline, the total HCit concentration ranged from 342 to 1763 µmol/mol, with a mean ± SD value of 684 ± 300 µmol/mol and a median [IQR] of 624 [509–788]. The protein-bound HCit concentrations ranged from 211 to 1675 µmol/mol, with a mean ± SD value of 557 ± 292 µmol/mol and a median [IQR] of 505 [393–603]. After 24 weeks of treatment, the mean ± SD total HCit and protein-bound HCit concentrations were stable (694 ± 445 µmol/mol and 544 ± 512 µmol/mol, respectively). The differences between total and protein-bound HCit concentrations at baseline and after 24 weeks of treatment were not significant (Fig. 1). Moreover, there were no significant differences in the HCit concentration between the nicotinamide and sevelamer groups.

**Fig. 2:**
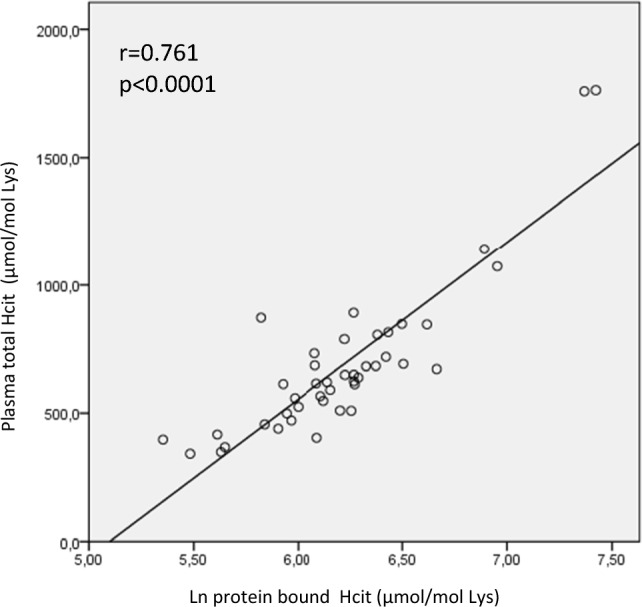
The relationship (in a linear regression) between baseline total HCit and protein bound HCit concentrations in hemodialyzed patients. *HCit* homocitrulline, *ln* logarithm. The data had a non-Gaussian distribution and so was log-transformed

**Fig. 3:**
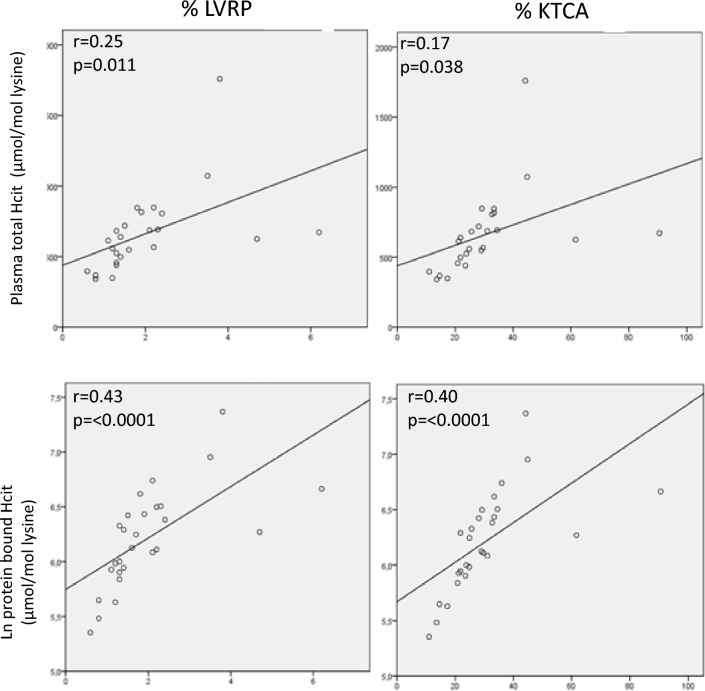
Linear regression between baseline albumin carbamylation levels and baseline total and protein-bound homocitrulline concentrations. *HCit* homocitrulline, *ln* logarithm, *LVRP and KQTA* the two albumin carbamylation sites. The data had a non-Gaussian distribution and so was log-transformed

The total HCit concentration was strongly correlated with the protein-bound HCit concentration (Fig. 2). The baseline total and protein-bound HCit concentrations were positively and significantly correlated with the degree of albumin carbamylation at the KQTA site (*r* = 0.174, *p* = 0.038, and *r* = 0.401, *p* < 0.0001, respectively) and at the LVRP site (*r* = 0.252, *p* = 0.01, and *r* = 0.427, *p* < 0.001, respectively) (Fig. 3). Similarly, the total and protein-bound HCit concentrations were significantly correlated (*p* < 0.0001) with the degree of albumin carbamylation after 24 weeks of treatment with either sevelamer or nicotinamide.

## Discussion

Our present results highlighted the presence of elevated HCit concentrations in hemodialyzed patients and also evidenced a significant correlation between HCit concentrations and albumin carbamylation levels at baseline and after 24 weeks of treatment with either sevelamer or nicotinamide. In both the sevelamer or nicotinamide groups, HCit concentrations did not decrease after 24 weeks of treatment (as previously shown for carbamylated albumin (Lenglet et al. 2017)); this might be explained by the small decrease in urea concentrations, which was probably not sufficient to lower CDP concentrations. HCit is considered to be a reliable biomarker of the overall carbamylation process, and the latter has been linked to various negative outcomes for hemodialyzed patients. Several studies have shown that urea has an indirect toxic effect via protein carbamylation, which interferes with the proteins’ properties and cellular functions and is associated with the progression of chronic kidney disease (Kalim et al. 2014). Furthermore, Koeth et al. (2013) found a significant association between elevated plasma HCit concentrations and death in hemodialyzed patients. Therefore, HCit might also be a valuable candidate biomarker of these negative outcomes. In recent years, several biomarkers of carbamylation have been described: carbamylated hemoglobin, carbamylated albumin, carbamylated lipoproteins, and carbamylated sortilin (Massy and Liabeuf 2022). In CKD patients, however, variability in the metabolism of components like hemoglobin has notably been linked to (i) changes in the lifespan of red blood cells, (ii) the prescription or not of erythropoietin, and (iii) blood loss during dialysis. Human albumin has a half-life of 3 weeks in plasma and has several sites that can be carbamylated (predominantly lysine 549). As a result, the percentage of carbamylated albumin represents a valuable biomarker of protein carbamylation—even though its half-life and concentration can be influenced by various pathophysiological conditions like liver failure and denutrition. Moreover, today’s methods for determining carbamylated albumin are technically complex and time-consuming; they require long preprocessing steps (including enzymatic digestion and solid-phase extraction) and specific technical skills for data analysis). Thus, the main objective of the present study was to quantify HCit in the NICOREN study, in order to determine whether or not (i) other carbamylated proteins contribute differently to protein carbamylation in this context and (ii) carbamylated albumin could be replaced by more easily assayed HCit as a marker of the overall protein carbamylated rate. Our present results showed that plasma protein-bound HCit is strongly correlated with carbamylated albumin. This correlation with plasma total HCit is weaker—probably because of the contribution of free HCit derived from the carbamylation of free amino acids (Kraus et al. 1998). Furthermore, assays of carbamylated albumin focus on specific sites, which might differ with regard to the kinetics of carbamylation. In contrast, HCit provides an estimation of the average overall carbamylation rate (i.e. including the carbamylation of other high-titer plasma proteins, such as immunoglobulins). A correlation between HCit and a specific carbamylated protein (hemoglobin) has already been evidenced (Jaisson et al. 2016), which confirms that HCit constitutes a good marker for the overall carbamylation process.

## Conclusion

Given the large number of candidate carbamylation biomarkers and the complexity of the corresponding specific analytical techniques, assaying plasma total and protein-bound HCit is less complex and less time consuming and provides information about the overall carbamylation process. HCit could therefore be considered as a biomarker of choice in forthcoming clinical studies of the role of carbamylation in ESKD.
